# A Reproducible and Scalable Process for Manufacturing a Pfs48/45 Based *Plasmodium falciparum* Transmission-Blocking Vaccine

**DOI:** 10.3389/fimmu.2020.606266

**Published:** 2021-01-11

**Authors:** Susheel K. Singh, Jordan Plieskatt, Bishwanath K. Chourasia, Amanda Fabra-García, Asier Garcia-Senosiain, Vandana Singh, Karin Lövgren Bengtsson, Jenny M. Reimer, Robert Sauerwein, Matthijs M. Jore, Michael Theisen

**Affiliations:** ^1^ Department for Congenital Disorders, Statens Serum Institut, Copenhagen, Denmark; ^2^ Centre for Medical Parasitology at Department of Immunology and Microbiology, University of Copenhagen, Copenhagen, Denmark; ^3^ PATH’s Malaria Vaccine Initiative, Washington, DC, United States; ^4^ Department of Medical Microbiology, Radboud University Medical Center, Nijmegen, Netherlands; ^5^ Novavax AB, Uppsala, Sweden

**Keywords:** malaria, vaccine, Pfs48/45, R0.6C, transmission-blocking, *Lactococcus lactis*, current Good Manufacturing Practices

## Abstract

The cysteine-rich Pfs48/45 protein, a *Plasmodium falciparum* sexual stage surface protein, has been advancing as a candidate antigen for a transmission-blocking vaccine (TBV) for malaria. However, Pfs48/45 contains multiple disulfide bonds, that are critical for proper folding and induction of transmission-blocking (TB) antibodies. We have previously shown that R0.6C, a fusion of the 6C domain of Pfs48/45 and a fragment of PfGLURP (R0), expressed in *Lactococcus lactis*, was properly folded and induced transmission-blocking antibodies. Here we describe the process development and technology transfer of a scalable and reproducible process suitable for R0.6C manufacturing under current Good Manufacturing Practices (cGMP). This process resulted in a final purified yield of 25 mg/L, sufficient for clinical evaluation. A panel of analytical assays for release and stability assessment of R0.6C were developed including HPLC, SDS-PAGE, and immunoblotting with the conformation-dependent TB mAb45.1. Intact mass analysis of R0.6C confirmed the identity of the product including the three disulfide bonds and the absence of post-translational modifications. Multi-Angle Light Scattering (MALS) coupled to size exclusion chromatography (SEC-MALS), further confirmed that R0.6C was monomeric (~70 kDa) in solution. Lastly, preclinical studies demonstrated that the R0.6C Drug Product (adsorbed to Alhydrogel^®^) elicited functional antibodies in small rodents and that adding Matrix-M^™^ adjuvant further increased the functional response. Here, building upon our past work, we filled the gap between laboratory and manufacturing to ready R0.6C for production under cGMP and eventual clinical evaluation as a malaria TB vaccine.

## Introduction

Malaria is a vector-borne disease caused by parasites of the *Plasmodium* genus. Of these, *Plasmodium falciparum* is causing the highest rates of morbidity and mortality worldwide with an estimated 229 million cases and 405,000 deaths globally in 2018 (1). Despite years of research, there is still no commercially available malaria vaccine. Until recently, the focus of malaria vaccine development has been to neutralize the parasite in infected individuals, but this approach has only been moderately successful (2–6). It may be more attractive to target the parasite in the mosquito as parasite numbers in mosquitoes are relatively low and form a bottleneck in the life-cycle of *P. falciparum*. Transmission blocking vaccines (TBVs) aim to induce antibodies that, together with the parasites in the bloodmeal, are taken up by the mosquito. Inside the mosquito midgut the antibodies inhibit parasite development blocking onwards transmission (7, 8). Proof-of-concept studies in humans that support this strategy have recently been published (9, 10).

Transmission of *P. falciparum* from one person to another depends on the production of male and female parasites, the so-called gametocytes that can be taken up by the mosquito while it is feeding on an infected individual (11). Once inside the mosquito midgut, the male and female parasites emerge from the erythrocyte as gametes and after a few rounds of replication, motile male gametes adhere to and penetrate female gametes to form zygotes. The *P. falciparum* surface protein Pfs48/45 is essential for the fertility of male gametes (12). A series of monoclonal antibodies (mAbs) have been generated against distinct epitopes of Pfs48/45 with the capacity to fully block parasite transmission in the Standard Membrane Feeding Assay (SMFA), which is the gold standard for assessing transmission blockage (13–16). Of these, mAb45.1 possesses the strongest TB activity and recognizes the conformational epitope I in the C-terminal Pfs48/45-6C domain (17).

Although Pfs48/45 is an attractive vaccine target, it has been difficult to produce a Pfs48/45 immunogen that is capable of eliciting functional antibodies in preclinical models [reviewed in (8)]. The challenges in the production of functionally active Pfs48/45 are most likely related to insufficient protein folding capabilities of employed expression systems. Correct folding of Pfs48/45 depends on proper disulfide bond formation, which is difficult to obtain in heterologous expression systems. The *Lactococcus lactis* expression-secretion system represented a significant advancement in the production of Pfs48/45 (18). Using this system, we have produced the C-terminal domain of Pfs48/45 (6C) containing the pertinent TB epitope I and three disulfide bonds, when expressed as a fusion protein (R0.6C) to the N-terminal region (R0) of the *P. falciparum* Glutamate-Rich-Protein (GLURP) (19, 20). We hypothesized that the intrinsically unstructured R0-domain serves as a carrier protein, which provides for the formation and subsequent stabilization of disulfide-bonds in this expression-secretion system (21). In order to ensure optimal folding of the final product, we have used the conformation-dependent and reduction-sensitive mAb45.1 to guide construct design and process-development. Accordingly, recombinant R0.6C has consistently generated specific antibodies in preclinical models with the capacity to inhibit parasite transmission in the SMFA (19, 20). One of the major challenges in the purification of functionally active R0.6C has been to separate correctly and incorrectly folded protein species. In proof-of-concept experiments, we previously immune-purified properly folded R0.6C on agarose-immobilized mAb45.1 (19) and more recently explored Ni-ion affinity chromatography for purification of R0.6C with a six-histidine tag (20). A histidine-tagged protein utilizes metal affinity chromatography (Nickle) which may pose potential limitations and hazards such as allergic reactions. Further the extraneous amino acids (six histidine) not native to the protein sequence, while suitable sometimes in initial safety and efficacy trials, are not recommended by regulators since they are not product related and serve no efficacy or immunogenicity purpose.

It is likely that a malaria TBV antigen will require a robust, scalable, and low-cost process that is easily transferrable among manufactures and countries to produce the required number of doses. In the first step of moving away from preclinical development work, this manuscript describes the process development of R0.6C without tags for purification, utilizing common and scalable column chromatography amenable to current Good Manufacturing Practices (cGMP). In doing so, we both optimized expression and improved recovery, thereby increasing our process yields, while also utilizing non-affinity chromatography strategies. The cycle-time (or overall process duration) was decreased by using a simple flow of column eluates to the subsequent column loads, without further buffer exchange or extensive processing steps. The overall quality of the antigen was either maintained or improved, in the respect of purity, integrity, identity, and process residuals, which are all important benchmarks in moving towards a viable vaccine candidate. Here, we detail the R0.6C expression and purification from bench-scale (1 L) through upscaling to support evaluation in Phase I clinical studies. To complement the strategy for the generation of Drug Substance presented here, we also present the planned Drug Product configuration (absorbed to Alhydrogel^®^).

## Methods

### Construct Preparation and Research Cell Bank (RCB)

Plasmid pSS105 harboring the gene for R0.6C without a his-tag (20) was transformed into *L. lactis* MG1363 and plated on solidified LAB medium (3.5% yeast extract, 0.05 mM FeSO_4_ 7 H_2_O, 2.5 μM CaCl_2_, 2.65 μM MgCl_2_, 0.5 mM citric acid 2H_2_O, 1.38 mM ammonium sulphate, 14.7 mM sodium-acetate, 20 mM KH2PO4 buffer pH 7.4) containing 1μg/ml erythromycin, 5% glucose, 4% di-sodium glycerophosphate, and 1.5% agar-agar. A clone was selected from two consecutive streaks for singles colonies. At each step, one single colony was selected based on its expression profile as determined by SDS-PAGE and used for the following streak. The clone was then obtained from the final streak and its ability to efficiently produce R0.6C was verified. A sterile flask containing 10 ml of LAB medium and 10 μg/ml erythromycin was inoculated with the clone selected above and incubated at 30°C. Following overnight incubation, 1.26 ml of the pre-culture was inoculate into a 250 ml flask containing 125 ml of LAB medium and 10 μg/ml erythromycin and incubated at 30°C. Cells were harvested at an OD_600_ of 0.8 to 1.0. Cells were centrifuged at 4,000 rpm for 15 min at 4°C, washed in 65 ml of LAB medium without erythromycin and centrifuged under the same conditions. Finally, the bacterial pellet was suspended in 25 ml of buffered LAB medium containing erythromycin (10 μg/ml) and 15% glycerol. The cell suspension was aliquoted aseptically into 2 ml polypropylene cryovials. The RCB was stored at -80°C. In-process controls were performed to assess purity and identity of the RCB.

### Fermentation at Bench-Scale and Sample Preparation

Fermentation of MG1363/pSS105 was performed as described previously (20) with minor modifications. Briefly, 5 ml pre-culture was grown at 30°C for 6h in LAB medium with 5% Glucose, 4% di-sodium glycerophosphate and 1µg/ml erythromycin by thawing one vial of frozen RCB. The fermenter (BIOFLO 310, New Brunswick Scientific) containing 1L LAB medium supplemented with 5% glucose, 5 mM cysteine, 0.5 mM cystine and 1 µg/ml erythromycin was inoculated with 0.4 ml pre-culture. After 4 h of inoculation, the fermenter was supplied with 50% glucose continuously at the rate of 8 ml/h to maintain 5% glucose in the medium until end of the fermentation. For the time-course experiment, 10 ml samples were withdrawn and used for analysis. Optical density at 600 nm (OD600) was used to assess cell density.

Cultivation was carried out at 30°C with gentle stirring (150 rpm) for 15–18 h until an OD600 of 12–15 was reached. After 18 h of growth, the bulk cell mass was removed by centrifugation (9,000×*g*, 4◦C, 30 min). The culture supernatant was concentrated 5-fold and buffer exchanged in buffer A (20 mM HEPES, 5%Glucose, 50 mM Sod. Borate, 10 mM L-arginine, 1 mM EDTA, pH6.5) using a QuixStand Benchtop system (Hollow fiber cartridge with cutoff at 30,000 Da, surface area 1,400 cm^2^, GE Healthcare, Sweden) followed by filtration through a Nalgene Rapid-Flow Sterile Disposable filter units with PES membrane 0.22 μm pore size.

### Protein Production at Bench Scale

The recombinant R0.6C protein was purified by sequential chromatographic separation using a 3-step procedure. 1) Capturing by IEC on a HiPrep Q HP column, 2) Removal of HCP on a HiPrep SP HP column, and 3) Isolation of the monomer fraction by IEC on a HiPrep Q HP column (GE Healthcare, Sweden). Protein concentration was measured by NanoDrop (A_280_) or by the BCA protein assay (Thermo Fisher Scientific) according to manufacturer’s instructions. Column chromatography was performed using a NGC 10 Medium Pressure Chomatography system (Biorad, USA).

#### Step 1. Capturing by Anion Exchange Chromatography

In the first step, R0.6C was captured from concentrated and buffer exchanged fermentation supernatant by anion exchange chromatography. The HiPrep Q HP column (GE Healthcare, Sweden) was equilibrated with buffer A (**Table 1**) in NGC 10 Medium Pressure Chromatography system (BioRad). The concentrated fermentation supernatant was passed over the charged column at a rate of 5 ml/min. After loading, the column was washed using equilibration buffer until the UV signal was reached up to baseline. Bounded R0.6C was eluted by using 200 mM NaCl buffer A (**Table 1**). The UV signal (280 nm) was used to automatically collect the elution peak into a 96 well plate (2 ml/well). The eluates were stored at 2–8°C until further processing.

**Table 1:** Column parameters for the purification of R0.6C.

IEC Q HP Capturing	1 L	5 L
Resin	HiPrep Q HP (GE Healthcare)	
Dimensions	Prepacked 16/10	XK-26/20
Flow rate	5 ml/min	5 ml/min
Column Volume	20 ml	79.6 ml
Column Pressure	0.5 Mpa	0.6 Mpa
Buffer A: 20mM HEPES, 5%Glucose, 50mM Sod. Borate, 10mM L-arginine, 1mM EDTA, pH6.5	Steps Load: 0% Wash: A+50 mM NaCl Wash: A+100 mM NaCl Elution: A+200 mM NaCl Strip: A+1M NaCl A	Steps Load: 0% Wash: A+50 mM NaCl Wash: A+100 mM NaCl Elution A+200 mM NaCl Strip: A+1M NaCl
Load volume	0.2 L	1.5 L
Load conductivity	7.49 mS/cm	7.79 mS/cm
Load pH	6.5	6.5
Eluate Volume	60 ml	240 ml
Eluate Conductivity	21.7 mS/cm	23.81 mS/cm
Eluate Peak Height	1,394 mAU	1,018.78 mAU
Eluate Peak Area	34,674	97,423
**IEC SP HP HCP removal**	**1 L**	**5 L**
Resin	HiPrep SP HP (GE Healthcare)	
Dimensions	Prepacked 16/10	XK-26/20
Flow rate	4 ml/min	4 ml/min
Column Volume	20 ml	79.6ml
Column Pressure	0.5 Mpa	0.6 Mpa
Buffer B: 20mM HEPES, 5% Glucose, 5mM L-arginine, 1mM EDTA, pH6.5	Steps Load: 0% Wash: B+50mM NaCl Wash: B+100mM NaCl Elution: B+150mM NaCl Strip: B+1M NaCl	Steps Load: 0% Wash: B+50mM NaCl Wash: B+100mM NaCl Elution: B+150mM NaCl Strip: B+1M NaCl
Load volume	480 ml	1920 ml
Load conductivity	4.48 mS/cm	4.15 mS/cm
Load pH	6.5	6.5
Eluate Volume	80 ml	320 ml
Eluate Conductivity	9.45 mS/cm	10.75 mS/cm
Eluate Peak Height	51 mAU	95 mAU
Eluate Peak Area	4034.2	7559
**IEC Q HP Polishing**	**1 L**	**5 L**
Resin	HiPrep Q HP (GE Healthcare)	
Dimensions	Prepacked 16/10	XK-26/20
Flow rate	4 ml/min	4 ml/min
Column Volume	20 ml	79.6 ml
Column Pressure	0.5 Mpa	0.6 Mpa
Buffer C: 20mM HEPES, 5% Glucose, 1mM EDTA, pH8.0	Steps Load: 0% Wash: C+150 mM NaCl Wash: C+270 mM NaCl Elution: C+310 mM NaCl Strip: C+1M NaCl	Steps Load: 0% Wash: C+150 mM NaCl Wash: C+270 mM NaCl Elution: C+310 mM NaCl Strip: C+1M NaCl
Load volume	480 ml	1920ml
Load conductivity	2.97 mS/cm	2.4 mS/cm
Load pH	8.0	8.0
Eluate Volume	26 ml	104 ml
Eluate Conductivity	25.7 mS/cm	25.54 mS/cm
Eluate Peak Height	97 mAU	110 mAU
Eluate Peak Area	1,155	1,520

#### Step 2. Removal of HCP by Cation Exchange Chromatography

The eluted fractions (from step 1) containing around 200 mM NaCl were diluted by a 1:8 ratio in buffer B (**Table 1**). In preparation for loading on to the HiPrep SP HP column (GE Healthcare, Sweden) column equilibrated with buffer B (**Table 1**) in the same machine as mentioned above. The pooled material was loaded on the HiPrep SP HP column at a rate of 4 ml/min and eluted by increasing the concentration of NaCl to 150mM in buffer B (**Table 1**).

#### Step 3. Isolation of Monomer Fraction by Anion Exchange Chromatography

Finally, the eluted material from the cation exchange chromatography (step 2) containing around 150 mM NaCl was diluted 1:6 in buffer C (**Table 1**) and loaded on to equilibrated HiPrep Q HP column (GE Healthcare, Sweden). Bounded R0.6C was eluted by adding 310 mM NaCl to buffer C (**Table 1**). The fractions containing monomers of R0.6C were pooled and diluted in formulated buffer (20 mM HEPES, 5% Glucose, 310 mM NaCl, and 1 mM EDTA, pH 8.0) and was stored at -80°C until further use.

### Process Scale Up

Fermentation process, sample preparation and protein purification at 5L scale was performed as described above at bench-scale. Here, we up scaled the column size of each steps of purification as mentioned in **Table 1**.

### SDS-PAGE and Immune Blotting

Samples were diluted with 6X SDS (Sodium dodecyl sulfate, Sigma-Aldrich) sample buffer, heated for 10 min at 98°C and loaded onto NuPAGE Novex 4%–12% Bis-Tris pre-cast gels (Invitrogen) according to the manufacturer’s instructions. Gels were run at 150–200 V for 35–50 min in 1X MOPS SDS running buffer and stained with Coomassie-staining procedures. Following SDS-PAGE proteins were transferred to nitocellulose membrane (BioRad) for immune analysis with conformational and reduction-sensitive Rat mAb 45.1 (1.0 μg/ml), and Rabbit anti-*L. lactis* anti-serum (1:500) or a *L. lactis* HCP kit (Cygnus technologies, USA) as previously described (20).

### Stability Study of R0.6C

A short-term and a long-term stability study of purified R0.6C was performed at different time points. For long-term stability, aliquots of R0.6C purified protein (Drug substance) were kept at -80°C for 0, 3, 6, 9, and 12 months. Protein degradation was analyzed by SDS-PAGE followed by western blot with mAb45.1 and protein aggregation by analytical SEC. For short-term stability, R0.6C was withdrawn from -80°C and incubated at 4°C, 25°C and 37°C for 0, 1, 2, 7, 14, 30, and 60 days. Samples were analyzed together.

### Analytical Methods

Analytical methods were developed for in-process characterization of R0.6C.

#### Kinetic Endotoxin Assay

Pierce LAL Chromogenic Endotoxin Quantitation Kit (Thermo Scientific) was used to quantify endotoxin content of purified proteins.

#### Molar Extinction Coefficient

The molar extinction coefficient (M-1•cm-1) was determined at 280 nm. The sample was serially diluted using the sample formulation buffer and the spectrophotometer was also autozeroed against the formulation buffer. The results are presented in terms of the nominal protein concentration (1 mg/ml) and on the basis of the protein concentration determined by amino acid analysis.

#### Residual DNA Content

Residual DNA was determined using 20 μl of sample by fluorometric assay using the Qubit^®^ dsDNA HS (High Sensitivity) Assay Kit from Life Technologies. The assay is highly selective for double-stranded DNA (dsDNA) over RNA and is sensitive down to 50 pg dsDNA (quantitation range 10 ng/ml to 100 µg/ml).

#### N-Terminal Sequence Analysis

50 μl of sample (710 pmol) was applied to a bioprene-treated prosorb cartridge and subjected to 10 cycles of automated N-terminal sequence analysis.

#### Amino Acid Analysis (AAA)

AAA was performed in triplicate using 30 μl of sample (30 µg) for each hydrolysis (6N HCl; 110°C; 20 h in sealed, evacuated glass tubes). Cysteine and Tryptophan were not determined.

#### Mass Spectrometry

Accurate molecular mass of R0.6C was measured by LC-ESI-MS under both non-reducing and reducing conditions as previously described (20).

#### Analytical Size-Exclusion High-Performance Liquid SEC-MALS, Dynamic Light Scattering (DLS)

SEC-MALS and DLS was performed as previously described in detail (22).

### Animals and Immunogenicity Studies

In the first immunogenicity study, groups of 5 female and 5 male CD-1 mice (Charles River, Germany) were used. In the second immunogenicity study, groups of 5 female CD-1 mice were used (Charles River, Germany). Mice were immunized intramuscularly in the right thigh with 50 µl vaccine, two times with a 4 weeks interval. Alhydrogel formulations contained 75 micrograms of Alhydrogel and were mixed by pipetting for 5 min. Matrix-M^™^ adjuvant (Novavax AB, Uppsala, Sweden) formulations contained 5 micrograms of Matrix-M adjuvant per injection and were mixed by pipetting for a short period of time. 70% Montanide ISA720 (Seppic, France) formulations were prepared following the manufacturer’s instructions. Formulations that contained both Alhydrogel and Matrix-M adjuvant were prepared by first adsorbing R0.6C to Alhydrogel as described above and then adding Matrix-M adjuvant. Fourteen days after the second immunization, mice were sacrificed and serum was collected for ELISA and SMFA analysis. All animal procedures complied with national regulations and were approved by the ethics committee of the Radboud University Medical Center (approval number 2016-0020).

### Enzyme Linked Immunosorbent Assay (ELISA)

Antibody responses in mice were measured by ELISA. 96-well plates (Nunc MaxiSorp) were coated with 0.5 μg/well SpyC-6C (23), incubated with serum, and bound antibodies were detected with HRP-conjugated polyclonal rabbit anti-mouse IgG (P0260, Dako). Antibody midpoint titer (EC_50_) was calculated using sigmoidal curve fitting. Statistical differences between same-dose different-adjuvant groups was determined by Mann-Whitney test. Reported p-values are 2-sided. Both curve fitting and statistical analyses were conducted in GraphPad Prism version 8.0.

### Standard Membrane Feeding Assay (SMFA)

The functional activity of sera was assessed in SMFA as previously described (24). Wild type *P. falciparum* NF54 gametocytes were mixed with human serum containing active complement and mouse serum (1:9 final concentration in blood meal) and fed to *Anopheles stephensi* mosquitoes. After 6–8 days, 20 fed mosquitoes were dissected and oocysts were counted after staining with mercurochrome. All sera were tested in two independent SMFA experiments. Transmission reducing activity (TRA) and statistical differences were determined using General Linearized Mixed Models (GLMMs) with zero-inflated negative binomial error structure using R version 3.6.2 (25, 26).

## Results

### Expression of R0.6C TBV Candidate in *L. lactis*

Plasmid pSS105 encoding the gene for R0.6C without a his-tag (20) was transformed into *L. lactis* MG1363 and plated on solidified LAB medium containing 1μg/ml erythromycin. A clone was selected from two consecutive streaks for singles colonies and used for the preparation of the Research Cell Bank (RCB). The RCB without his-tag was then utilized to develop a robust downstream workflow independent of affinity (lacking monoclonal antibodies or Ni-ions) columns.

### Fermentation at Bench Scale

**Figure 1:**
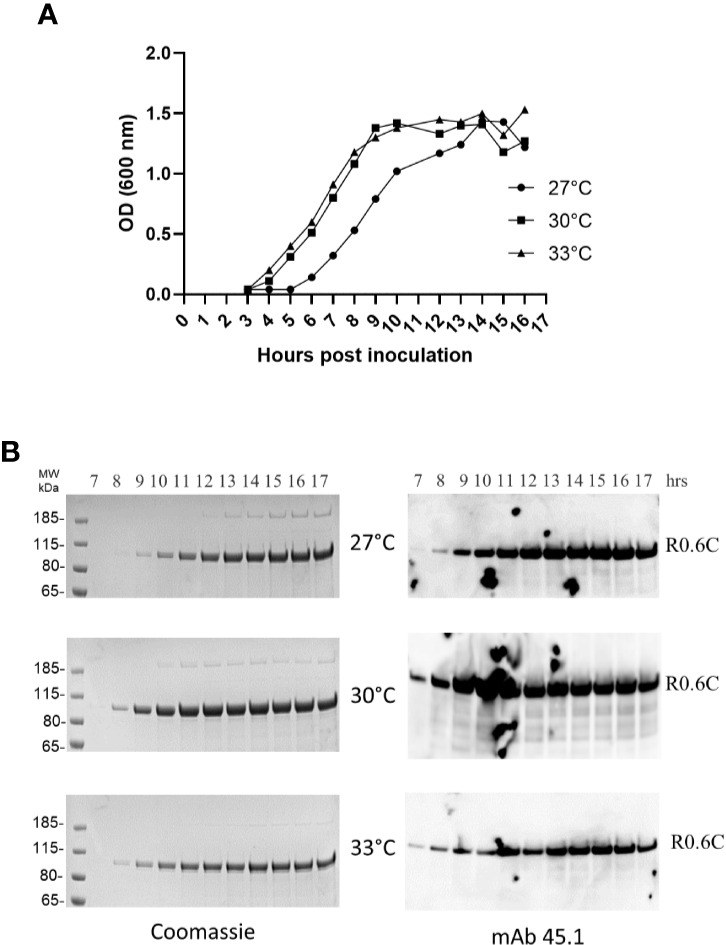
Time course analysis of R0.6C expression in *L.lactis* at different temperatures. **(A)** Optical density measurements at 600 nm from 3 to 17 h of bacterial growth at 27, 30, and 33°C. **(B)** Samples collected from 7 to 17 h were assessed by Coomassie and immune blotting analysis. Samples were analysed without a reducing agent. *Left panel*, Coomassie blue-stained 4%–12% polyacrylamide gel. *Right panel*, immune blotting analysis of the same gel shown to the left using the conformational reduction-sensitive mAb45.1 as primary antibody.

Fermentation of MG1363/pSS105 at 27, 30, and 33°C demonstrated the extracellular production of R0.6C increased through 16 h of growth and produced up to 55–60 mg of R0.6C per liter culture supernatant. (**Figure 1A**). The results indicate that bacterial growth rate as well as quantity and quality of secreted R0.6C was identical at all temperatures tested (**Figures 1A, B**). Analysis of culture supernatants by SDS-PAGE analysis and immune blotting with mAb45.1, which recognize epitope I located in the 6C-domain of the R0.6C fusion protein, indicated that the fermentation process is robust and that R0.6C was produced as a properly folded full-length protein (**Figure 1B**).

### Purification of R0.6C at Bench Scale

#### First Step

**Figure 2:**
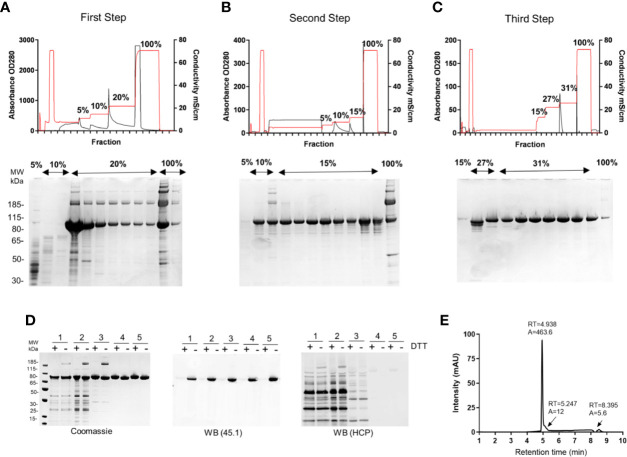
Production of R0.6C in *L. lactis*. Elution profiles of R0.6C on **(A)** First step; HiPrep Q HP column, **(B)** Second step; HiPrep SP HP column, and **(C)** Third step; HiPrep Q HP. Selected fractions were analysed on 4%–12.5% polyacrylamide gels shown below the chromatograms. Protein was loaded without a reducing agent. **(D)** Analysis of R0.6C by SDS-PAGE. *Left panel* Coomassie blue-stained 4%–12.5% polyacrylamide gel; 1 Fermented supernatant 2 Diafiltrate, *3* HiPrep Q HP column, *4* HiPrep SP HP column, 5HiPrep Q HP column purified R0.6C. An immune blot analysis of the same gel shown in the *left panel* using mAb45.1 and anti-*L. lactis* antibodies (detection of HCP) in the *middle* and *right panels*, respectively. Protein was loaded in each lane with (+) or without (−) DTT (10 mM). The sizes (kDa) of the molecular mass markers are indicated. **(E)** Reverse-phase (RP) HPLC. Reversed-phase HPLC–UV chromatogram recorded following analysis of purified R0.6C. The peak at 4.938 min corresponds to monomeric R0.6C antigen. A denotes integrated peak area, and RT indicates retention time.

The first step was optimized to capture 80%–90% of the target antigen, R0.6C, with minimal binding of unwanted HCP. Due to the high content of Glutamic residues in the GLURP-R0 portion of the R0.6C fusion protein (19) a series of anion exchange resins were screened for the capturing of R0.6C. It was found that R0.6C binds tightly to Q-Sepharose at pH 6.5 and conductivity below 10 mS/cm. To ensure maximum recovery from the capturing step, the loading density was maintained at 20 mg/ml of resin or less. The clarified culture-supernatant was applied to a HiPrep Q-HP (16/10) column, washed with 5 column volumes (CVs) of 50 mM NaCl in Buffer A followed by 8 CVs of 100 mM NaCl in the same buffer (**Table 1**). Bound material was eluted with 10 CVs of 200 mM NaCl and fractions containing the desired protein (**Figure 2A**) were pooled and diluted 8-fold with 20 mM HEPES, 5% Glucose, 5 mM L-arginine, 1 mM EDTA, and pH 6.5 (**Table 1**) to bring the conductivity below 5 mS/cm for application to the subsequent column. Buffer components were previously screened to aid in the stability and purification of R0.6C: 1) HEPES was selected as the buffer system as phosphate may interfere with adsorption to Alhydrogel^®^, 2) Glucose as a stabilizer to prevent aggregation 3) L-arginine to maintain proper folding, and 4) EDTA to prevent degradation during the purification process. Under these conditions, the efficiency of the capture step was approximately 45–50 mg/L R0.6C, or approximately ~ 80% (**Table 2**).

**Table 2:** Mass Balance summary of R0.6C.

Steps	1 L			5 L		
	Total protein (mg/L)	R0.6C (mg/L)	Step Recovery^d^ (%)	Total protein (mg/L)	R0.6C (mg/L)	Step Recovery^d^ (%)
Harvest Supernatant	23,500^a^	55–60	N/A	22000^a^	55–65	N/A
Diafiltration/buffer Exchange	750	55–60	100	670	53–55	96
Capture (Q)	215	45–50^b^	81	195	45–50^b^	85
HCP removal (SP)	59	40–45^b^	72	55	35–40^b^	77
Polishing (Q)	32	25–27^c^	62	30	23–25^C^	65

^a^Total protein is over-estimated due to the presence of Cysteine and Cystine in the medium.

^b^Amount of R0.6C in the eluate.

^c^Amount of R0.6C monomer in the eluate.

^d^Recovery defined as percentage of R0.6C recovered from prior step.

#### Second Step

The diluted eluate from the capturing step was then applied onto a HiPrep SP HP (16/10) column at 5 mg of protein per ml of resin at 4ml/min. The column was washed with 5 CVs of 50 mM NaCl followed by 8CVs of 100 mM NaCl in Buffer B and bound protein was eluted with 10 CVs of 150 mM NaCl in the same buffer (**Table 1**). Fractions containing high concentration of R0.6C were pooled resulting in a mixture of monomeric and multimeric forms of R0.6C (**Figure 2B**). The overall efficiency of this step was approximately 85% (**Table 2**), yielding 40–45 mg/L R0.6C. To prepare the protein sample for binding to the final anion exchange polishing column, it was important to reduce the conductivity to <5 mS/cm by diluting 6-fold dilution with Buffer C (**Table 1**).

#### Third Step

Multimeric R0.6C does not elicit TB antibodies possibly because intermolecular disulfide bonds disrupt folding of the conformational TB epitope I in the 6C-region (20, 21). Therefore, a last polishing step was included to obtain pure monomeric R0.6C. We have previously demonstrated that Q Sepharose has the capacity to separate the monomeric and multimeric forms of R0.6C (20). The diluted eluate from the second column was therefore applied again to a HiPrep Q-HP (16/10) column at 3 mg/ml of resin. The column was washed with 5 CVs of 150 mM NaCl followed by 8 CVs of 270 mM NaCl in Buffer C thereby removing residual HCPs and smaller MW protein fragments resulting from proteolytic degradation of the target protein (**Figure 2C**). Bound protein was eluted with 10 CVs of 310 mM NaCl (**Table 1**). Fractions containing a single band of monomeric R0.6C were pooled resulting in a final yield of approximately 25–27 mg/L **R0.6C** (**Table 2**), which equals approximately 50% overall recovery of monomeric R0.6C yielded from the culture supernatant. The purity and folding of each step as determined by SDS-PAGE and immune blotting with mAb45.1 and anti-*L. lactis* antibodies (detection of HCP) are shown in (**Figure 2D**, **Table 3**). The purity of the final R0.6C was also assessed by reversed-phase HPLC. There R0.6C eluted as a main peak at 4.938 min (**Figure 2E**). The presence of few minor peaks indicates a homogenous population of R0.6C protein with a relative purity of 97.6% (**Table 3**).

**Table 3:** Characterization of R0.6C.

Test	Method	Result			
		Consistency 1(1L)	Consistency 2(1L)	Consistency 3(1L)	Lab-Scale(5L)
pH	pH meter	8.0	8.0	8.0	8.0
Color and Appearance	Visual observation	Colorless	Colorless	Colorless	Colorless
Molar Extinction Coefficient (M^-1^•cm^-1^)	spectroscopy	13, 913.4^a^			ND^b^
Protein Content	A_280_/Nanodrop	0.7 mg/ml	0.8 mg/ml	0.75 mg/ml	1.1 mg/ml
Accurate quantification of the total protein	AAA analysis	0.72 mg/ml	ND	ND	1.2 mg/ml
Endotoxin	LAL	17 EU/mg	8.0 EU/mg	11 EU/mg	6.0 EU/mg
Residual host-cell DNA	Qubit Fluorescence Assay	2.7 pg/µl^a^			
N-terminal sequencing analysis	N-terminal sequencing	AERSTSENRN^a^			ND^b^
Residual host-cell protein by ELISA	Immuno-enzymatic Assay	<1%	<1%	<1%	<1%
Identity and Integrity (SDS-PAGE)	SDS-PAGE both non-reduced and reduced	Major monomer band	Major monomer band	Major monomer band	Major monomer band
Identity (WB)	Non-reduced and reduced Western Blot against monoclonal antibodies 45.1	Major monomer band	Major monomer band	Major monomer band	Major monomer band
Purity (RP-HPLC)	RP-HPLC	97.6%	>97.3%	>96.9%	>95%
Identity, Integrity and Purity (SE-HPLC)	SE-HPLC	98.5% monomer	97.9% monomer	98.2% monomer	95.5% monomer
Intact Mass spectrometry analysis of full-length protein (LC-MS/MS)	Intact mass spec under non-reducing and reducing conditions	Non-reduced (70257.906Da) ^a^ Reduced (70263.852Da) ^a^			Non-reduced (70257.916Da) Reduced (70263.863Da)

^a^A single sample from the three runs were utilized for contracted testing. Consistency run#1 solely analyzed.

^b^ND, Not determined or performed on sample.

### Protein Analysis by Mass Spectrometry

**Figure 3:**
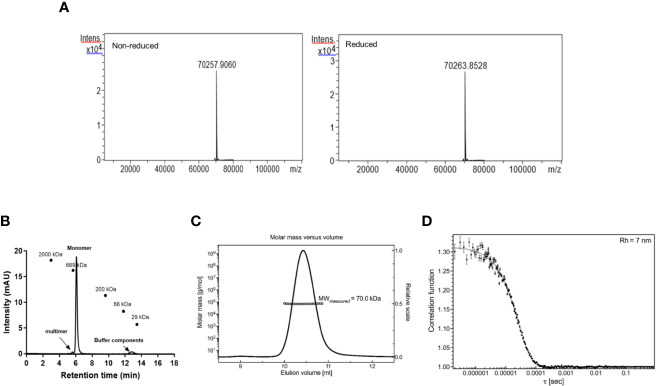
Characterization of R0.6C. **(A)** deconvoluted mass spectra corresponding to the non-reduced and reduced samples. **(B)** Size-exclusion chromatography (SEC) HPLC analysis. SEC-HPLC analysis was performed under native conditions in a phosphate buffer pH6.7 to determine the amount of monomer in the sample. The peaks observed at +40 min are due to salts in the buffer matrix. **(C)** SEC-MALS analysis. R0.6C was injected onto a Superdex 200 Increase 10/300GL column and the change in refractive index as a function of protein concentration was used to compute the molar masses. The solid line plotted on the *right axis* corresponds to the change in refractive index from the SEC column. The right axis is normalized to the greatest magnitude across the chromatogram data, i.e., to the monomer peak of R0.6C. The molar masses across the eluting peak are plotted as open circles on the *left axis* (molar mass). The average molecular mass is indicated. **(D)** QELS goodness of fit of the auto-correlation function plot at the apex of the peak.

The identity of the R0.6C was confirmed by mass-spectrometry-based methods. The molecular mass of non-reduced R0.6C was 70,257.90 ± 2 Da as determined by LC–MS (**Figure 3A**, left panel). This molecular weight corresponds well to the predicted value of 70,257.90 assuming that recombinant R0.6C contains the vector encoded amino acid residues A-E-R-S at the N-terminal end. These results indicate that R0.6C does not contain any post-translational modifications and that the secreted, soluble protein was intact. Reduction of R0.6C resulted in an increase measured mass of 6 Daltons to 70,263.85 ± 2 Da, which is consistent with 3 disulfide bonds (**Figure 3A**, right panel, **Table 3**). Thus, the R0.6C protein mainly contain all six cysteine residues in the oxidized state.

### Protein Characterization

**Figure 4:**
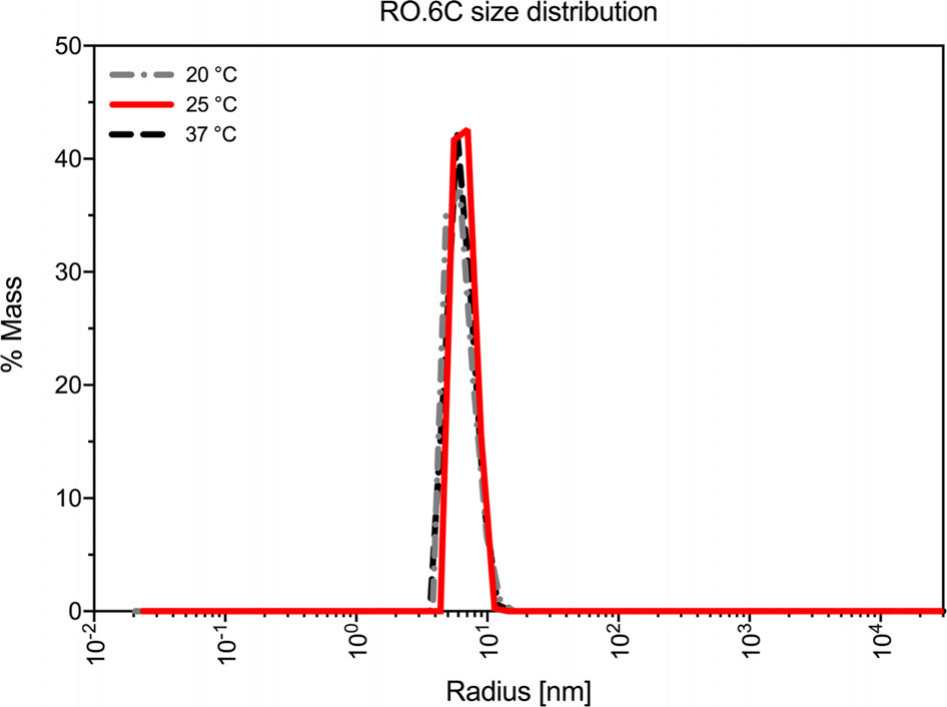
Biophysical studies of R0.6C. Dynamic light scattering (DLS) proﬁle of the R0.6C at three different temperatures.

Purified R0.6C was analyzed by SEC-HPLC to determine the monomer content of the final material. Protein eluted in multiple peaks (**Figure 3B**, **Table 3**), of which the Peak at 6.079 contains the majority of the sample. The calculated MW was approximately 600 kDa based on a range of globular proteins included in the molecular weight standard. The very large MW estimate suggests that R0.6C either forms large aggregates in solution or has a very large hydrodynamic volume due to structural properties (21). Multi-Angle Light Scattering (MALS) coupled with size exclusion chromatography (SEC-MALS) was used to determine the absolute molecular weight of soluble R0.6C without the use of reference proteins. R0.6C eluted as a main monodisperse peak at 10.5 ml (**Figure 3C**) with an average molecular mass of 70.0 kDa indicating that R0.6C is a monomer in solution. Indeed, disorder prediction indicated that the R0-region of R0.6C is intrinsically disordered (21), providing a possible explanation for the aberrant migration pattern by SEC of a larger molecular weight. Furthermore, the hydrodynamic radii (Rh) of the monomeric recombinant R0.6C proteins were examined by QELS. The Rh of R0.6C was 7.0 nm (**Figure 3D**), indicated the monomeric form of R0.6C is highly intrinsically disordered and extended compared with atypical globular protein. To analyze the behavior of R0.6C proteins in solution, in particular to probe for aggregation and stability the dynamic light scattering (DLS) was performed. R0.6C consists of a homogenous population of mainly monomeric protein species with a low percentage of polydispersity (<25%) and an average size of radius 7.7 nm and diameter 15.4 nm in both pre and post thermal elevation (**Figure 4**). R0.6C was also further analyzed by N-terminal sequencing to confirm the N-terminus, amino acid analysis (AAA) and absolute extinction coefficient (**Table 3**). The sequence determined, NH2-AERSTSENRN, exactly matches the predicted sequence of R0.6C.

In addition, residual DNA content, endotoxin and host cell protein was also assessed supporting that the overall purification process yielded material below the acceptable limits (**Table 3** and **4**).

**Table 4:** R0.6C current Good Manufacturing Practices (cGMP) proposed release assays.

Attribute	Test	Analytical test method	Proposed specifications
Biochemical	Appearance	Visual Assessment USP <1790>	Clear, colorless solution free of particles
Biochemical	pH	Potentiometric pH USP <791>	7,5–8,5
Biochemical	Total Protein Concentration	SE-HPLC	≥ 0.4mg/ml
Biochemical	Endotoxin	LAL	≤ 100 EU/mg
Identity	Protein ID	Western blot	Positive identification and predominant band at expected molecular weight
Identity/Purity	R0.6C Full-length MW	SDS-PAGE	Predominant band at expected molecular weight ≥ 85%
Identity/Purity	R0.6C Full-length	SE-HPLC	≥ 85% monomer
Potency	Biological Activity	ELISA Folding	≥ 85%
Residual/Purity	Residual DNA	Quant-it	10 pg/µl to 100 ng/µl
Residual/Purity	Host cell protein	Lactococcus WB	<1% w/w
Residual/Purity	Boron/borate	ICP-AES	TBD
Safety	Pyrogen	Rabbit pyrogen testing USP <151>	USP <151>
Safety/Purity	Microbial enumeration	Bioburden USP <61>	USP <61>

### Stability Studies

**Figure 5:**
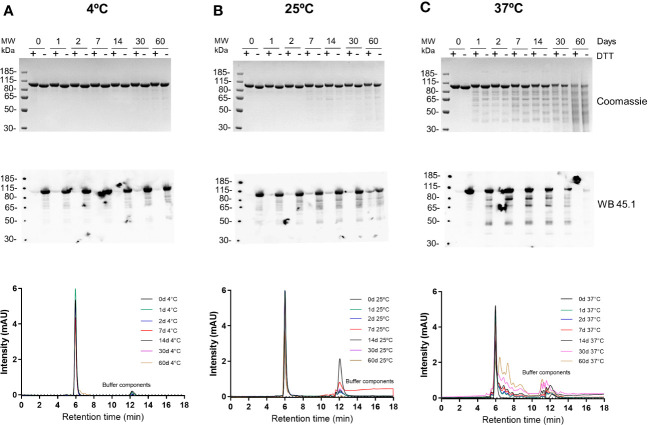
Stability analysis of R0.6C. Short term stability at **(A)** 4°C, **(B)** 25°C, and **(C)** 37°C; Coomassie blue-stained 4%–12.5% polyacrylamide gel; Numeric zero,1,2, 7,14, 30, and 60 corresponds to days at different temperatures(*upper panel)*;immune blot analysis of the same gels with mAb45.1 and size-exclusion chromatography (SEC) analysis (overlap chromatograms) of samples used (*middle and lower panel* respectively). Representative SEC chromatograms of R0.6C protein eluted contains the majority of the monomer.

**Figure 6:**
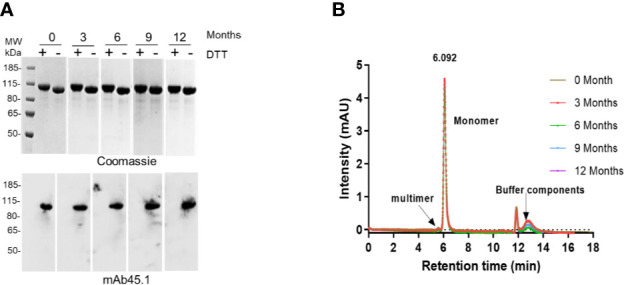
Long term stability at -80°C. **(A)** Coomassie blue-stained 4%–12.5% polyacrylamide gel; numerics Zero, 3, 6, 9, and 12 correspond to months (*upper panel*); an immune blot analysis of the same gel using mAb45.1 (*lower panel*). **(B)** SEC analysis (overlap chromatograms) of samples used in **(A)** Representative SEC chromatograms of R0.6C protein eluted contains the majority of the monomer.

The stability of the R0.6C protein was evaluated for 60 days at 4°C, 25°C, and 37°C (**Figure 5**). R0.6C showed little degradation after 60 days at 4°C and 25°C as judged by SDS-PAGE and immune blotting with mAb45.1 (**Figures 5A, B**, upper and middle panel). Moreover, the SEC-HPLC analysis of the same samples confirmed that the R0.6C purified protein does not form aggregates in solution at these temperature (**Figures 5A, B**, lower panel). In contrast, R0.6C protein did show signs of degradation after 2 days at 37°C (**Figure 5C**). The R0.6C protein showed no signs of neither degradation nor aggregation when stored at -80°C for 12 month (**Figure 6**) and stability analysis is ongoing.

### Process Consistency and Stability

To demonstrate the consistency of the production process, each step of the upstream and downstream processes was conducted three times at the 1 L scale. The banding pattern of the raw culture supernatants (**Figure S1A**) and diafiltrates (**Figure S1B**) were similar among the 3 runs, including the ratio of monomer/multimer and the HCP content (non-mAb45.1 reactive bands seen at lower molecular weights) is similar among runs as assessed by visual inspection of the Coomassie stained SDS-PAGE gels. Most of R0.6C was captured on the HiPrep Q-HP (16/10) and more than 80% of the host cell protein (HCP) was removed in this step (**Figure S1C**). The remaining HCP and putative R0.6C degradation products were removed in the HCP removal step on the HiPrep SP HP (16/10) column (**Figure S1D**). The monomer/multimer ratio remain unaffected by these purification steps. Monomer and multimer forms are separated during the last purification step resulting in the final product of pure monomeric R0.6C (**Figure S1E**). The immunoblot analysis of all the steps including the final purified product from the 3 consistency runs show similar reactivity with mAb45.1 demonstrating that the final upstream and downstream processes consistently gives a R0.6C product of the same folding and therefore biological activity. A characterization of the resulting purified R0.6C from the three consistency runs is summarized in **Table 3**. The summary of critical parameters of the final downstream process at bench scale is given in **Table 1**. Process hold steps were evaluated by looking at supernatant, Diafiltrate, Capturing (IEC Q HP column), HCP removal (IEC SP HP column) and Polishing (IEC Q HP column) at 2-8^0^C daily through seven days shows no change (**Figure S2**).

### Scale Up to 5 L

The bench-scale process was scaled up 5-fold and run in a manner identical to how the process would be performed in the GMP suite after further scaling to 30 L. For the purification process, column dimensions and conditions for 5 L scale were outlined in **Table 1**. The fermentation and protein yield per litre and protein purity was identical to that described above for the 1L scale **Tables 1**–**3**.

### Preclinical Studies

**Figure 7:**
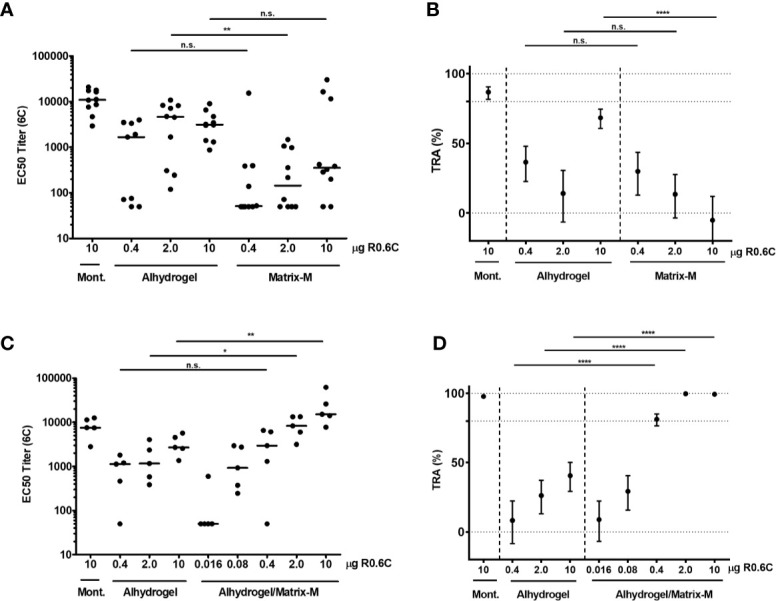
Immunogenicity of R0.6C vaccine formulations. Two separate mice immunization experiments were conducted with R0.6C formulated with different adjuvants. In each experiment one group received R0.6C formulated with Montanide (Mont.), which has previously been shown to induce high antibody titers (20). 6C-specific antibody titers for individual mice in the first **(A)** and second **(C)** experiment are shown as mid-point titers. Mid-point titers below 50 are reported as 50. Bars represent median values. Statistical difference between same-dose groups is determined by Mann-Whitney test, and reported p-values are two-sided (n.s. not significant, *p < 0.05, **p < 0.01). Pooled sera from the first immunization **(B)** and second immunization **(D)** were tested in Standard Membrane Feeding Assay (SMFA) at 1:9 dilution. Reported values and 95% confidence intervals (bars) are determined by General Linearized Mixed Models with zero-inflated negative binomial error structure and used oocyst count data from two independent SMFA experiments with 20 mosquitoes per condition and experiment. Transmission reducing activity is calculated by comparing to a non-serum control included in each SMFA. Statistical difference is determined between same-dose groups (n.s. not significant, ****p < 0.001).

We have previously shown that R0.6C, formulated with Alhydrogel, elicits transmission blocking antibodies in rats (19, 20). To identify more potent formulations, R0.6C with Matrix-M™ adjuvant, which is a saponin-based adjuvant composed of purified saponin from the tree *Quillaja Saponaria Molina* (27), was evaluated in mice. We used two immunizations instead of three that we previously used, to allow differentiation between potent and less potent adjuvants as well as reducing study duration to be consistent with planned vaccine potency development assays. Outbred CD-1 mice were immunized intramuscularly on days 0 and 28 with different dosages of R0.6C adjuvanted in either Alhydrogel^®^ or Matrix-M adjuvant. Two weeks after the last injection, mice were bled, and antibody titers and functionality of antibodies were assessed by ELISA and the SMFA, respectively. To measure 6C-specific antibodies, a plate antigen (SpyC-6C) with a unique carrier protein sequence (SpyC) was used. Matrix-M adjuvant did not induce higher 6C-specific antibody titers than Alhydrogel (**Figure 7A**). Pooled serum from mice that were immunized with 10 µg R0.6C with Alhydrogel, but none of the Matrix-M adjuvant groups, reduced transmission >50% in SMFA (**Figure 7B**). We next investigated whether addition of Matrix-M adjuvant to an Alhydrogel formulation could enhance immunogenicity in mice. 6C-specific antibody titers were significantly higher when mice were immunized with Alhydrogel/Matrix-M and 2 or 10 µg R0.6C, compared to groups that received equal amounts of R0.6C with Alhydrogel only (**Figure 7C**). Interestingly, pooled sera from groups that received Alhydrogel/Matrix-M and 0.4, 2, or 10 µg R0.6C reduced transmission >80%, which was significantly higher than the groups that received the same dose with Alhydrogel alone (**Figure 7D**). Altogether we show that a formulation of R0.6C on Alhydrogel/Matrix-M induces high levels of transmission reducing antibodies, warranting further testing of this formulation in humans.

## Discussion

We present the process development and scale-up of the malaria vaccine candidate R0.6C in the *L. lactis* expression-secretion system. Lactic acid bacteria have a well-established role in the production of fermented foods and more recently, they have gained an importance in the production of pharmaceuticals (28–30). The *L. lactis* expression-secretion system was used partly because nearly all other expression systems assessed so far have failed to produce a recombinant Pfs48/45 protein which elicit functional TB antibodies (reviewed in (8)) and partly because this system has proven effective for the production of other malaria vaccine candidates such as GMZ2 (31). Further, the *L. lactis* expression-secretion system i) does not produce endotoxins or extracellular proteases, ii) does not exhibit unwanted post translational modifications such as glycosylation, and iii) is “generally recognized as safe” (GRAS status) by the by the US Food and Drug Administration (FDA). The use of *L. lactis* is therefore an excellent alternative strategy for recombinant protein production. In addition, the cGMP compline manufacturing of R0.6C further strengthens its application in protein secretion for vaccine immunogen development.

The production of cysteine-rich proteins requires optimization of fermentation conditions to facilitate proper disulfide bond formation (19, 20, 22). This is particularly important aspect of the fermentation process as protective TB antibodies often target conformational epitopes. By carefully adjusting the ratio of the cysteine-cystine redox couple in the fermentation broth, we have established conditions that render the *L. lactis* extracellular milieu more oxidizing thereby enhancing formation of structural disulfide bonds in the Pfs48/45-6C domain of R0.6C. Using these conditions, properly folded monomeric R0.6C represents >60% of the total amount of protein secreted into the growth media. Notably, we confirmed that neither bacterial growth rate nor yield of properly folded monomeric R0.6C was affected by small temperature fluctuations proving that the lab-scale fermentation process is robust and amenable to tech-transfer and scalability. A secretion yield of approximately 60 mg/L seen at lab-scale is among the best obtained with this host organism and may be further improved by bioprocess optimization. It is evident that more insight into how different fermentation conditions affect protein production and folding is required. For this purpose, whole-genome sequencing, transcriptomics, and proteomics approaches are needed (32–34).

A downstream process was developed at bench-scale sufficient in respect to yield, purity, consistency, and potency while maintaining an overall ~50% recovery. One of the major obstacles in the purification of Pfs48/45-based vaccine antigens has been to isolate properly folded conformers without relying on immune-purification (20). Here, we have developed a simple workflow consisting of common ion-exchange chromatography with a capturing-step on a Sepharose Q HP column, followed by a second step on Sepharose SP HP column which removes HCP and other host cell contaminates such as residual DNA. The final polishing step served to separate monomeric and multimeric conformers of R0.6C. This final purification step is particularly important, as the multimeric protein species does not elicit functional TB antibodies (8, 18). Because proper folding of R0.6C depends on the formation of three disulfide bonds in the C-terminal Pfs48/45-6C domain, we used the activity of the disulfide reduction sensitive and conformational mAb45.1 to monitor the purification of properly folded protein species (18). Tight cuts of the product peak in the final polishing step were made to ensure the desired quality was maintained at the expense of yield. The robustness of the bench scale process was assessed through three consistency runs with identical upstream and downstream processes. The average yields from the three bench scale runs were ~25 mg/L culture broth. While the overall recovery is ~50%, such yields are sufficient for initial clinical evaluation and offer room for further later improvement if successful in initial clinical evaluations.

The analytical testing of the products from these consistency runs is presented in **Table 3** with planned cGMP Drug Substance release assays presented in **Table 4**, which are currently being qualified. The purity of R0.6C was 98.5% and the content of monomeric protein was 97% or greater by RP and SEC HPLC, respectively. Intact mass analysis of reduced and non-reduced R0.6C confirmed the expected MW and the presence of three disulfide bonds in the final product. The state of R0.6C in solution was further characterized by a variety of biochemical and biophysical methods. Accordingly, R0.6C proved stable for 7 days at 37°C as determined in the accelerated stability program. Detailed biophysical studies including DLS, SEC-MALS, and QELS experiments demonstrated that soluble R0.6C forms a homogenous population with mainly a monomeric protein species. Collectively, these results indicate that a consistent and robust process at the bench-scale and formed a solid starting point for tech-transfer and the technical specifications for the release tests for the cGMP product. The final product from the 3^rd^ consistency run served as a reference for process scale up and manufacturing.

The 5L scale as presented here serves as a basis for the intended 30L engineering and cGMP manufacturing of R0.6C. Overall column dimensions and parameters are detailed in **Table 1** which are then easily scaled to accommodate 30L upstream processes using standard scale-up approaches. The average overall yield for the process scale-up runs and the purity by RP and SEC HPLC was comparable to those obtained at bench-scale. The process as described is capable to produce 25 mg of properly folded monomeric R0.6C per liter of fermentation broth, with the potential for yields to increase during scale-up. Assuming a dose of 100 µg R0.6C, the yield is 7,500 doses from the 30L cGMP run. This yield is clearly sufficient for the planned phase 1 clinical trials and likely sufficient for Phase II as well. The best way to substantially increase yields is by scaling the fermentation volume; there are no requirements for oxygen and vigorous stirring during fermentation so scaling up to 200–1,000 L would be simple and straightforward. Alternatively, the biomass may be increased by removing lactate from the culture broth during fermentation through systems such as for example the REED™ (Reverse Electro-Enhanced Dialysis) technology (35). Future clinical evaluations in human volunteers will provide initial data, which will determine whether further process optimization and scale-up are required.

We have previously demonstrated that R0.6C formulated with Alhydrogel induces functional responses in rodents (19, 20). Although this is an attractive formulation to test in humans given its widespread use and longstanding safety profile, we also set out to identify more potent formulations, as Alhydrogel alone may not elicit the high antibody titers required for a malaria TBV. We (36, 37) and others (38, 39) have previously demonstrated that immunogenicity of protein based formulation may be improved by the addition of adjuvants. The Matrix-M adjuvant technology (40) is a promising technology which has been explored for various infectious diseases and has a good safety profile in humans (27, 41–43). Further, our studies were designed to make use of a single R0.6C Drug product (Alhydrogel formulation) which serves as a suitable and safe adjuvant for first in human clinical studies of novel antigens, while also maintain the ability to added additional adjuvants to the same drug product to increase the immunogenicity should it be needed. Here, we have used a suboptimal vaccination schedule to detect differences in immunogenic properties between vaccine formulations. R0.6C formulated with either Alhydrogel or Matrix-M adjuvant failed to induce high levels of functional antibodies in mice. In contrast, the addition of Matrix-M to R0.6C adsorbed on Alhydrogel substantially increased the immunogenicity of R0.6C resulting in functional antibodies even at the low dose of 0.4 µg R0.6C. While the study presented here does not utilize the same species and immunization regimen as previous (19, 20), we demonstrate that the non-tagged R0.6C antigen developed here can induce a consistent and protective immune response with human compatible adjuvants, as well as reducing the variability among the dose response from individual animals. These studies lay the groundwork for future potency and biological assay development for the R0.6C TBV candidate that will be utilized in cGMP release and stability. Although it is not yet fully understood how the Matrix-M adjuvant achieves its stimulatory effects, this adjuvant is known to transiently enhance the number of activated immune cells in the draining lymph nodes which may in turn lead to increased uptake and presentation of vaccine antigens to elicit a competent immune response (44). Specifically, it has been shown that there is an increase of CD169+ macrophages, as well as activated dendritic cells, to the draining lymph nodes after immunization with Matrix-M adjuvanted vaccines, which may help to increase antigen presentation (45). Hence, CD169+ macrophages has previously been shown to have a role in transporting antigens to B lymphocytes by trapping them in the draining lymph node and to facilitate cross-presentation of the antigen to CD8+ T lymphocytes (46, 47). This may lead to increased humoral and cellular immune responses, manifested by cross-reactive antibodies and multi-functional CD4+ T lymphocytes (41, 48). Consequently, Matrix-M has been shown to contribute to antigen dose-sparing and increased duration of humoral and cellular vaccine responses (42). Whether the Matrix-M adjuvant has a similar effect on R0.6C adsorbed on Alhydrogel remains to be investigated.

In conclusion, we have developed a process that can be used to generate cGMP grade R0.6C with proper cysteine connectivity. Importantly, we have identified two R0.6C formulations that elicited high levels of functional antibodies in preclinical models. Together our results pave the way for first time in-human testing of a Pfs48/45-based vaccine.

## Data Availability Statement

The original contributions presented in the study are included in the article/**Supplementary Material**. Further inquiries can be directed to the corresponding author.

## Ethics Statement

All animal procedures complied with national regulations and were approved by the ethics committee of the Radboud University Medical Center (approval number 2016-0020).

## Author Contributions

SS, JP, RS, MJ, KB, JR, and MT contributed conception and design of the study. SS, BC, AF-G, AG-S, VS, and MJ performed the experiments. JP and MT wrote final manuscript. All authors contributed to the article and approved the submitted version.

## Funding

This work was supported by grants from the European Union’s Horizon 2020 research and innovation program under grant agreement No. 733273, the European and Developing Countries Clinical Trials Partnership (RIA2018SV-2311), and by PATH’s Malaria Vaccine Initiative under Grant OPP1108403 from the Bill & Melinda Gates Foundation and in part by Grant NNF14CC0001. The funders had no role in the study design, data collection and analysis, decision to publish, or preparation of the manuscript.

## Conflict of Interest

The authors declare that the research was conducted in the absence of any commercial or financial relationships that could be construed as a potential conflict of interest.
