# Supplementary material for: A Reproducible and Scalable Process for Manufacturing a Pfs48/45 Based Plasmodium falciparum Transmission-Blocking Vaccine
Source: Front Immunol. 2021 Jan 11;11:606266. doi: 10.3389/fimmu.2020.606266 (PMC7832176; doi:10.3389/fimmu.2020.606266)
Supplement: Supplementary file 1 [file DataSheet_1.pdf]

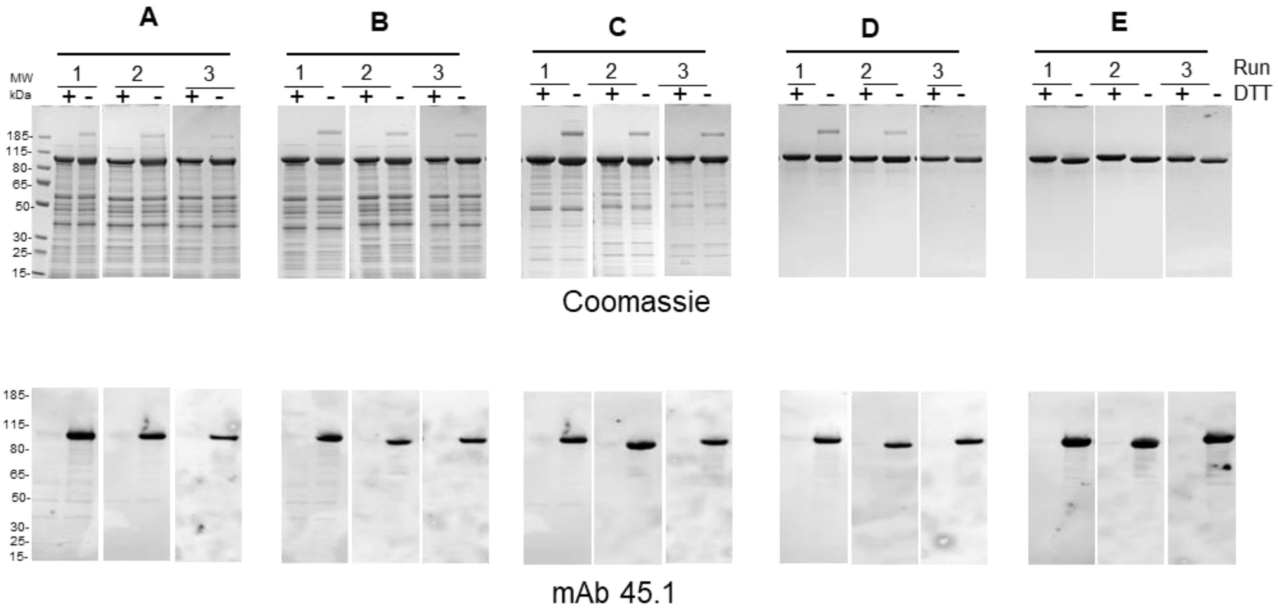

Figure S1. SDS-PAGE analysis of each step of consistency runs. *upper panel* Coomassie stained 4-12.5% polyacrylamide gel of **(A)** Supernatant (run 1-3), **(B)** Diafiltration (run 1-3), **(C)** eluate from capturing step (run 1-3), **(D)** eluate from HCP removal step (run 1-3), and **(E)** the final product the eluate from the polishing step (run 1-3). *Lower panel* an immune blot analysis of the same gel shown in the upper panel using mAb45.1as primary antibody.

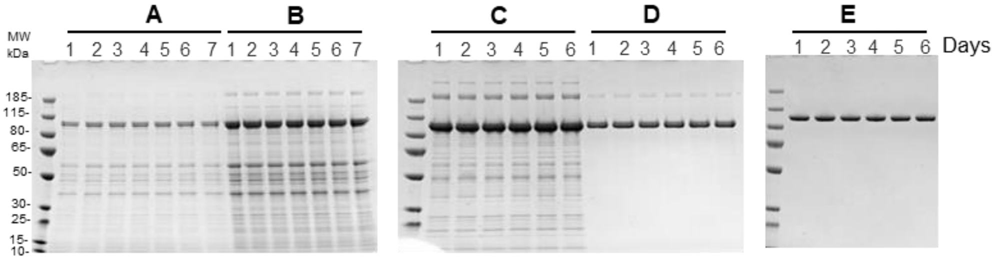

Figure S2. R0.6C stability during purification. R0.6C protein does not degrade when held at 2-8oC for 7 days. Coomassie blue-stained 4-12.5% polyacrylamide gel of fermentation and different purification steps. (A) Supernatant; (B) Diafiltrate; (C) Capturing (IEC Q HP column); (D) HCP removal (IEC SP HP column); (E) Polishing(IEC Q HP column).
